# Epiphytic bryophyte biomass estimation on tree trunks and upscaling in tropical montane cloud forests

**DOI:** 10.7717/peerj.9351

**Published:** 2020-06-12

**Authors:** Guan-Yu Lai, Hung-Chi Liu, Ariel J. Kuo, Cho-ying Huang

**Affiliations:** 1 Department of Geography, National Taiwan University, Taipei, Taiwan; 2 Department of Civil and Environmental Engineering, University of California, Los Angeles, Los Angeles, CA, USA; 3 Research Center for Future Earth, National Taiwan University, Taipei, Taiwan

**Keywords:** Conifer, Diameter at breast height (DBH), Lichen, Liverwort, Moss, Scaling, Taiwan, Tree size

## Abstract

Epiphytic bryophytes (EB) are some of the most commonly found plant species in tropical montane cloud forests, and they play a disproportionate role in influencing the terrestrial hydrological and nutrient cycles. However, it is difficult to estimate the abundance of EB due to the nature of their “epiphytic” habitat. This study proposes an allometric scaling approach implemented in twenty-one 30 × 30 m plots across an elevation range in 16,773 ha tropical montane cloud forests of northeastern Taiwan to measure EB biomass, a primary metric for indicating plant abundance and productivity. A general allometry was developed to estimate EB biomass of 100 cm^2^ circular-shaped mats (*n* = 131) with their central depths. We developed a new point-intercept instrument to rapidly measure the depths of EB along tree trunks below 300 cm from the ground level (sampled stem surface area (SSA)) (*n* = 210). Biomass of EB of each point measure was derived using the general allometry and was aggregated across each SSA, and its performance was evaluated. Total EB biomass of a tree was estimated by referring to an in-situ conversion model and was interpolated for all trees in the plots (*n* = 1451). Finally, we assessed EB biomass density at the plot scale of the study region. The general EB biomass-depth allometry showed that the depth of an EB mat was a salient variable for biomass estimation (*R*^2^ = 0.72, *p* < 0.001). The performance of upscaling from mats to SSA was satisfactory, which allowed us to further estimate mean (±standard deviation) EB biomass of the 21 plots (272 ± 104 kg ha^−1^). Since a significant relationship between tree size and EB abundance is commonly found, regional EB biomass may be mapped by integrating our method and three-dimensional remotely sensed airborne data.

## Introduction

Bryophytes are rootless, non-vascular terrestrial plants such as mosses, liverworts and hornworts. Due to their primitive physiological characteristics, bryophytes are sensitive to the recent changes in climate such as increases in air temperatures (Aptroot & Van Herk, 2007; Zotz & Bader, 2009) and atmospheric carbon dioxide (Turetsky, 2003), and decreases in precipitation (Gignac, 2001). Epiphytic bryophytes (EB) are species that grow on the surface of a plant above the ground. They are some of the most representative lifeforms of tropical montane cloud forests (TMCF) (Barkman, 1958; Smith, 1982), which are ecosystems that experience frequent immersion of low altitude cloud (also known as “fog”, exchangeably used hereafter) with high humidity. Tropical montane cloud forests, as suggested by their name, are mostly distributed over mountainous regions. While covering only about 0.14% (~30 M ha) of the Earth’s terrestrial surface (Bruijnzeel, Mulligan & Scatena, 2011) and 2.5% of tropical forests of the world (Bubb et al., 2004), they provide major water sources for lowland environments due to the orographic effect and cloud water interception (Bruijnzeel, Mulligan & Scatena, 2011). As a result, TMCFs play a disproportionately-large role in the functioning of a global terrestrial ecosystem relative to their limited distribution.

Epiphytic bryophytes may obtain necessary water and nutrients for growth by intercepting parallel precipitation (fog water) (Stadtmüller, 1987; Holwerda et al., 2010; Scholl, Eugster & Burkard, 2011). In some regions, EB are keystone species for providing water and essential nutrients to maintain the health of TMCFs (Gradstein, 2008; Zotz & Bader, 2009) and may affect carbon storage of an entire ecosystem. They may also influence the global hydrological cycle by modifying precipitation and evaporation levels (Rhoades, 1995; Chang, Lai & Wu, 2002; Porada, Van Stan & Kleidon, 2018). In recent decades, land use and land cover have changed (Ray et al., 2006), and the prevailing global trend of elevated temperatures (Still, Foster & Schneider, 1999; Foster, 2001) may alter regional climate in tropics, resulting in substantial ramifications on EB (Benzing, 1998) and eventually TMCF. As “canaries in the coal mine” (Gignac, 2001), spatiotemporal dynamics of EB may be effective indicators for monitoring regional and global climate changes. One of the very first steps in this research field is to quantify the abundance of EB, which has been a very challenging task due to nature of their habitats and diverse morphologies (McCune & Lesica, 1992).

Biomass is a major metric to assess the abundance of plants (Bonham, 2013). For EB, biomass is also a key indirect parameter to assess the capacity of TMCFs to intercept fog for storing, intercepting and slow releasing water and nutrients (Zotz & Vollrath, 2003; Chen, Liu & Wang, 2010; Kürschner & Parolly, 2004; Ah-Peng et al., 2017; Rodríguez-Quiel, Mendieta-Leiva & Bader, 2019). The abundance of EB in TMCFs may be affected by microclimatic (e.g., humidity, temperature, luminosity) and host structural (such as tree size, height and density) attributes (Peck, Hong & McCune, 1995; Freiberg & Freiberg, 2000; Nöske et al., 2008; Chen, Liu & Wang, 2010). Field survey approaches such as destructively sampling with interpolation on the ground for low stature (Ah-Peng et al., 2017) or fallen (Chen, Liu & Wang, 2010) trees, and using a ladder, rope (Hsu, Horng & Kuo, 2002; Nakanishi et al., 2016), high tower or crane (McCune et al., 1997; McCune et al., 2000) to reach tall trees have been commonly implemented to measure EB biomass (see Table 1 a comprehensive summary). However, field EB measurements have been known to be quite challenging to carry out, which made regional quantification impractical (Moffett & Lowman, 1995; Barker & Pinard, 2001). In this article, we proposed a simple and effective field allometric scaling method to estimate EB biomass for TMCF, which combines small-scale destructive field biomass collection, vertical point intercept sampling conducted by a newly-invented instrument, and upscaling the biomass estimation with a previously established in-situ equation and data interpolation. Our specific research questions follow:

Q_1_: Can we estimate EB biomass at the patch scale using a single structural parameter?
Q_2_: Can we upscale the patch scale estimation to the whole tree scale?
Q_3_: What is the feasibility of the proposed approach for the plot scale EB biomass estimation and the potential for the regional spatial assessment?

**Table 1:** Summary of the plot or the forest stand scale epiphytic bryophyte biomass density (kg ha^-1^) research reported in the refereed literature. For the sake of quality, only peer-reviewed articles are listed. The table is organized based upon the data collection methods; “Climbing” includes the use of rope or ladder, and “Ground” indicates EB samples were reachable from the ground or removed from fallen logs. We note that studies that combined terrestrial bryophyte biomass or did not specify the collection of EB biomass only are not listed in this table. Annual precipitation (AP, mm y^−1^), mean annual temperature (MAT, °C) and elevation (m a.s.l.) of each site were directly obtained from its corresponding article. If the information was missing, it was then obtained from the internet. The ecosystems labeled as TMCF could be tropical montane cloud forest, or other similar forest ecosystems including tropical montane rain forest or tropical montane moist forest. The ones categorized as TCF are temperate conifer forests. To make the comparison legitimate, dead EB and humus mass was not included in the estimation. Studies only sampled part of EB biomass of trees such as a tree trunk (e.g., Kürschner & Parolly, 2004) are also not listed here.

Method	Location	AP	MAT	Elevation	Ecosystem	Tree sample	EB biomass	References
Climbing	La Soufriére, Guadeloupe	1,780	26.3	1,330	TMCF	Not available	12,336	Coxson (1991)
	Mascarene Archipelago, Madagascar	8,000	24	1,350	TMCF	Not available	9,020	Ah-Peng et al. (2017)
	Santa Rosa de Cabal, Colombia	1,250	5.5	3,700	TMCF	1	6,850	Hofstede, Wolf & Benzing (1993)
	Olympic Mountains, US	4,700	9.6	179	TCF	3	6,527	Nadkarni (1984a)
	Cordillera de Talamanca, Costa Rica	5,193	16.8	1,555	TMCF	15	6,225	Köhler et al. (2007)
	Monteverde, Costa Rica	2,591	18.6	1,480	TMCF	25	4,058	Nadkarni et al. (2004)
	Cordillera de Talamanca, Costa Rica	2,812	10.9	2,900	TMCF	6	1,921	Hölscher et al. (2004)
	Fushan, Taiwan	3,600	18.2	750	TMCF	18	1,740	Hsu, Horng & Kuo (2002)
	Monteverde, Costa Rica	2,591	18.6	1,700	TMCF	4	945	Nadkarni (1984b)
	Northeast China	1,450	−0.8	875	TCF	Not available	507	Ye, Hao & Dai (2004)
	The Tilaran Range, Costa Rica	5,380	17.7	1,325	TMCF	6	206	Häger & Dohrenbusch (2011)
Harvesting	Monteverde, Costa Rica	2,591	18.6	1,480	TMCF	9	2,087	Nadkarni et al. (2004)
	Yunnan, China	1,931	11.3	2,500	TMCF	77	1,663	Chen, Liu & Wang (2010)
	Cordillera Oriental, Colombia	1,850	6	3,650	Bamboo	Not available	1,281	Tol & Cleef (1994)
	Rwenzor Mountains, Uganda	2,000	8.5	3,230	TMCF	1	1,000	Pentecost (1998)
	Marafunga Basin, New Guinea	3,985	13	2,625	TMCF	42	940	Edwards & Grubb (1977)
	Zamora Chinchipe, Ecuador	2,080	15.5	2,093	TMCF	63	604	Werner et al. (2012)
	Central French Guiana	2,500	27	288	TMCF	15	452	Gehrig-Downie et al. (2011)
	Cascade Range, US	2,450	9.2	655	TCF	42	323	McCune (1993)
Ground	Southern Thailand	2,000	28.5	804	Tropical forests	51	126	Chantanaorrapint & Frahm (2011)
	North Wales, UK	2,187	10.3	98	TCF	16	87	Rieley, Richards & Bebbington (1979)
Scaling	Chilan mountain, Taiwan	3,500	12.7	1,680	TMCF	210	272	This study

## Materials and Methods

### Study site

The study was focused on 16,773 ha TMCFs of Chilan Mountain (24°98′N, 120°97′E) in northeastern Taiwan (the spatial boundary defined by referring to Schulz et al., 2017) administered by the Veterans Affairs Council, R.O.C. (field permit number: 1080002884). The precipitation in summer and winter consists of mostly orographic precipitation and tropical cyclones (regionally known as typhoons), and the northeastern monsoon, respectively. Annual precipitation and mean temperature of the site are 3,500 mm y^−1^ and 12.7 °C, respectively (Wang & Huang, 2012; Hu & Huang, 2019). The mean (± standard deviation (SD)) elevation of the site is 1680 ± 343 m a.s.l., and mean slope (± SD) is 38.2° ± 13.4° ranging from 0° to 88.7°. The rugged terrain faces regular moist wind from the Pacific Ocean resulting in frequent occurrences of upslope fog approximately 300+ days of a year and 38% of the time (Lai et al., 2006). This humid bioclimate harbors a substantial amount of EB. There were 49 and 24 species observed in mature old-growth and regenerated forests, respectively, by a preliminary local inventory (Chang, Lai & Wu, 2002). The primary vegetation type of TMCFs is a relatively homogeneous conifer forest dominated by old-growth and regenerated hinoki cypress (*Chamaecyparis obtusa* var. *formosana*) stands with some Japanese cedar (*Cryptomeria japonica*) plantations. Shrubs are not common and no lianas are present in the mid-latitude TMCFs (Scatena et al., 2010). Bryophytes are the dominant group of the epiphytic substrate of the region, occupying 93.5% of the total biomass (Teng, 2006).

### The patch scale EB biomass sampling and model development

The first step was to derive a general allometry for EB biomass. Since elevation can be a crucial factor in the distribution of EB biomass abundance (Horwath et al., 2019), six sites within the elevation range of 1,200–1,950 m a.s.l. were randomly selected for sample collection (Fig. S1) in the summer (May–October) of 2017. The center depth (from rhizoids to the top of a plant) of each EB species within a 100 cm^2^ circular mat was recorded using a stainless steel ruler. There were 113 liverworts, 17 mosses and 1 lichen (for details of the species see the spreadsheet in Supplemental Information) measured. We note that one lichen sample was included in the model development to account for the presence of a small portion of lichen among EB. For every EB sample, we confirmed that there was only a single species (no mixture of species) in the circular mat with homogeneous depth before measuring the central depth. Each EB sample was measured from a randomly selected tree stem (total 131 host trees) within a reachable height of a person with a portable short ladder (e.g., 300 cm above the ground). Finally, the samples were removed using a gardening shovel. This method has been applied previously by Rodríguez-Quiel, Mendieta-Leiva & Bader (2019). The samples were stored in sealed linear low-density polyethylene bags to maintain moisture, then placed in an ice box and transported to a laboratory within 8 h after their removal from host trees. The samples were cleaned of dead organic matter, suspended soil and tree bark with tap water, dried in a 70 °C biomass oven for at least 72 h, and weighed using a three-decimal-place electronic balance (LIBROR EB-430H, Shimadzu, Japan). In this study, EB biomass was defined as the total sampled dry weight divided by the projected surface area of the sample (g cm^−2^). The depth of EB was used as a unique trait for each independent sample to develop EB biomass allometric equations:

(1) $$W={\rm \alpha}D^{\rm {\beta}}$$

*W* is the EB biomass density (g cm^−2^), *D* is the EB depth (cm), and α and β are the exponent components for the model (e.g., as intercept and slope after the log–log transformation to a linear representation). A power model was selected to fit the data as the nature of allometry in biology (West, Brown & Enquist, 1999) by referring to previous studies (Niklas, 1993, 2006) using “*nlme*” package (Pinheiro et al., 2019) in R v. 3.5.0. (R-Core Team, 2019). The Akaike information criterion (AIC), the Bayesian information criterion (BIC) and log-likelihood were considered when facilitating model selection (Burnham & Anderson, 2004). Consecutive values ranging from 0.01 to 2.0 with an interval of 0.01 were selected for β with and without a fixed α value of 1 to derive an optimized model to fit the empirical data using nonlinear least squares. In addition, the method generalized nonlinear least squares specifically designed to minimize the effect of unequal variances, which were commonly observed in ecological data (Pinheiro & Bates, 2006) was also utilized. Three variance covariate functions, the exponential of a variance covariate (varExp in R), power of a variance covariate (varPower) and constant plus power of a variance covariate (varConsPower), were used to modify regression of the fitted values and the residuals within the fitted models.

### The tree scale EB biomass estimation

The main goal of this study was to implement a new field method for the estimating and upscaling of EB biomass in TMCFs. Once the allometric model (Eq. (1)) had been established, the next step was to estimate EB biomass of a tree, and we could then interpolate the estimate. Twenty-one 30 × 30 m plots covering old growth, regenerated and plantation forest stands (Hu & Huang, 2019) (mainly hinoki cypress) along the elevation gradient of 1,260–1,990 m a.s.l. in Chilan Mountain of northeastern Taiwan were surveyed (Fig. S1). Diameter at breast height (DBH) measured at 130 cm above the ground for each living tree with DBH ≥ 5 cm within 16 and 5 plots was recorded in July of 2016 and January of 2019, respectively. We then selected 10 trees within each plot (total 210 trees) evenly distributed along the DBH gradient to interpolate EB biomass. Basal diameter (BD) of each sampled tree at the ground level was also measured, and the relationship between basal area and DBH was investigated.

**Figure 1:**
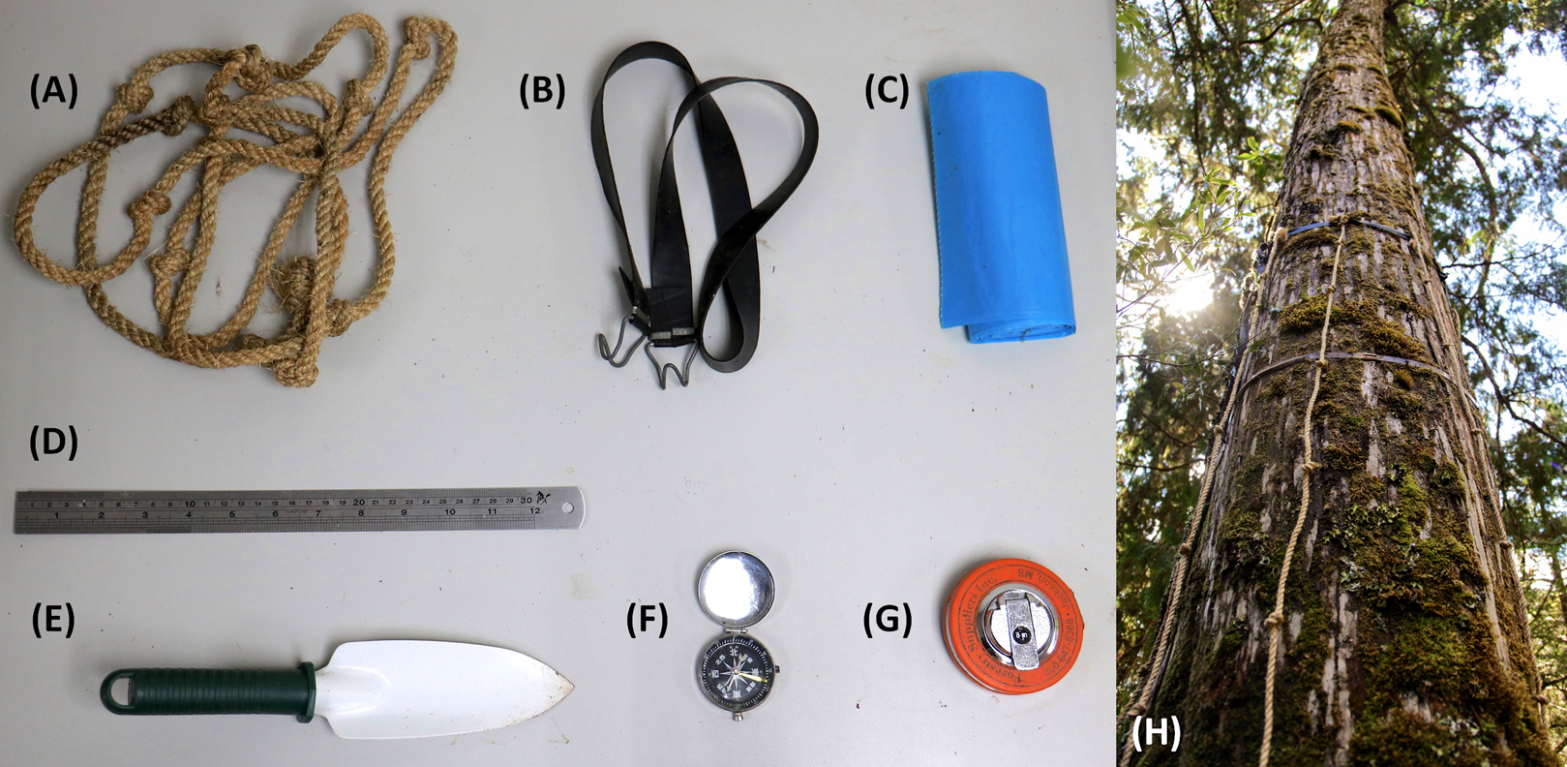
The field instrument utilized in this study to estimate the biomass of epiphytic bryophytes in tropical montane cloud forests of northeastern Taiwan. (A) A 3-m rope with 30-cm-long intervals marked by knots, (B) an adjustable rubber strip to fix ropes to a tree stem, (C) large, strong, and tear-resistant plastic bags to store EB from sampled stem surface area, (D) a stainless steel ruler to measure the heights of EB mats before removing samples with (E) a gardening shovel, (F) a compass to facilitate placing ropes in different orientations, (G) a fabric diameter tape to measure the sampled stem surface area. (H) A demonstration. The photograph was taken on Chilan Mountain by Guan-Yu Lai in January 2019.

According to Johansson (1974), Köhler et al. (2007) and a local study (Teng, 2006), the majority of EB (in their cases, 71–91%) were present at the lower part of a tree in TMCF, which may be a salient variable in upscaling EB biomass of an entire tree. Therefore, a new field instrument was designed specifically for the estimation of EB biomass at the stem/trunk or tree scale (Fig. 1). Depths of EB (including the absence of EB recorded as depths of 0 cm) were recorded for every 30 cm vertical interval in several directions from the ground to 300 cm of each sampled tree main stem height. The sampled trees with DBH larger than 20 cm were recorded in eight directions (north, northeast, east, southeast, south, southwest, west and northwest) otherwise in just four major cardinal directions (north, east, south and west) by referring to a compass. The Tukey–Kramer multiple comparison (“*stats*” in R) (Fox & Weisberg, 2019) with the significant level (α) of 0.05 was applied to compare the EB depths of different orientation groups. The data were converted to biomass density by referring to the allometry (Eq. (1)), and then averaged. We note that all trees in the plots were taller than 300 cm. Sampled stem surface area (SSA) of each tree was determined by measuring DBH and BD. Based on visual inspection, the shape of the trunk from the ground to 130 cm was defined as a truncated cone and from 130 cm to 300 cm from the ground as a cylinder. Accordingly, the surface area (cm^2^) of the trunk below 300 cm (SSA) was calculated by referring to Eqs. (2) and (3):

(2) $${\rm SSA} = 170\; \times \pi \times {\rm DBH} + \; \pi \times l\; \times \left( {\displaystyle{{\rm BD} \over 2} + \displaystyle{{\rm DBH} \over 2}} \right)$$

(3) $$l = \; \sqrt {{{130}^2} + {{\left( {\displaystyle{{\rm BD} \over 2} - \displaystyle{{\rm DBH} \over 2}} \right)}^2}}$$

where SSA (cm^2^), *l* (cm), DBH (cm) and BD (cm) are sampled stem area, slant length of the cone, diameter at breast height and basal diameter, respectively. The biomass of EB below 300 cm ($M_{\rm Total}^{\rm Tree}$) was then calculated by multiplying average biomass density by SSA for each sampled tree. In August 2019, we stripped EB mats of SSA from 30 randomly selected and widely-distributed trees of different sizes to verify the estimation.

### EB biomass up-scaling

The biomass of EB of 10 sampled trees of each plot was estimated by referring to Eq. (4):

(4) $$\ln \left( {M_{\rm Total}^{\rm Tree}} \right) = \; 0.99{\rm ln}\left( {\rm DBH} \right) + 0.68{\rm ln}\left( {M_{\rm SSA}^{\rm Tree}} \right) - 1.195,\quad R^2 = 0.99\,\,(p\,\lt\,0.001)$$

where $M_{\rm Total}^{\rm Tree}$ and $M_{\rm SSA}^{\rm Tree}$ are EB biomass (kg) of total surface area and SSA of a tree, respectively, according to the in-situ destructive measurement by stripping EB samples with a sampling interval of 0.5 m from 10 hinoki trees (mean DBH ± SD = 15.8 ± 6.4 cm ranging from 6.2 cm to 24.5 cm; mean tree height ± SD = 10.5 ± 1.9 m from 7.1–12.8 m) at the elevation of 1670 m a.s.l. (Teng, 2006). Epiphytic bryophytes of different heights were destructively sampled by chaining (eight trees) or ladder and rope climbing (two trees), and the procedure was similar to Bates (1982), Freiberg & Freiberg (2000) and Hsu, Horng & Kuo (2002). An apparent negative trend of tree height and EB biomass was observed (Fig. S2). Since the intercept of Eq. (4) is negative, resulting in negative values for small trees, a fixed ratio of 1.3 was then applied according to Teng (2006) for those trees. We realize that host tree species could affect EB species distribution and abundance due to differences in bark characteristics such as texture, water absorption capacity and/or pH (Studlar, 1982). However, this factor should be negligible in our case since the forest types are quite homogeneous in our study plots dominated by hinoki cypress. Sampled stem area of all trees ($SSA_{\rm Total}^{\rm Plot}$) in a plot was then estimated with the knowledge of DBH and DBH-BD of each tree (Eqs. (2) and (3)). Finally, EB biomass ($M_{\rm Total}^{\rm Plot}$, kg) (Eq. (5)) and its density (kg ha^−1^) of a plot may be estimated by referring to Eq. (5) with the knowledge of EB biomass ($M_{\rm Sampled}^{\rm Plot}$) (from Eq. (4)) and SSA ($SSA_{\rm Sampled}^{\rm Plot}$) of 10 sampled trees.

(5) $$\displaystyle{{{SSA}_{\rm Total}^{\rm Plot}} \over {{SSA}_{\rm Sampled}^{\rm Plot}}} = \; \displaystyle{{M_{\rm Total}^{\rm Plot}} \over {M_{\rm Sampled}^{\rm Plot}}}$$

Literature search was conducted in the largest academic search engine Google Scholar (https://scholar.google.com/) with the keywords “epiphytic bryophyte” and “biomass” for a general comparison of EB biomass density (with basic bioclimatic information). We note that for the sake of quality control, non-refereed articles such as graduate theses and conference proceedings were excluded.

## Results

### Epiphytic bryophytes biomass allometry

**Figure 2:**
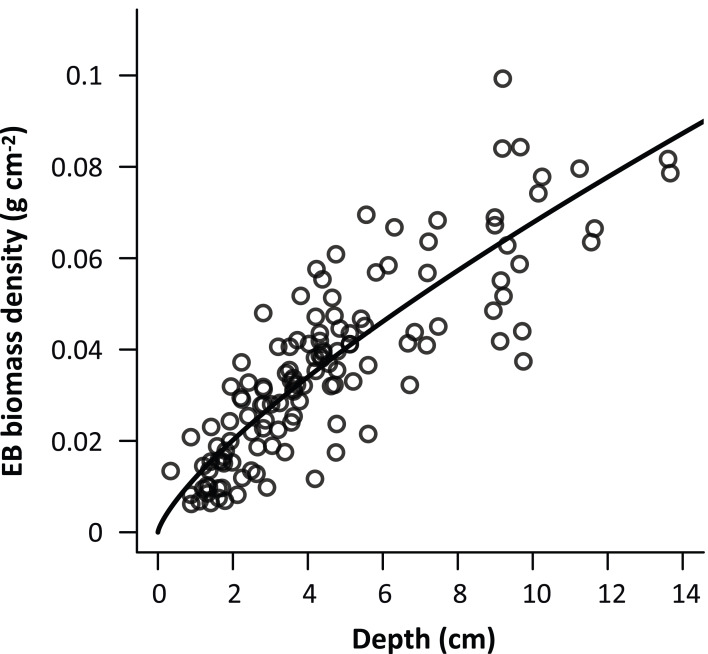
The best empirical general depth-biomass allometric model of epiphytic bryophytes. The best empirical general depth-biomass allometric model of epiphytic bryophytes (EB). The model was a power of variance covariate function (*R*^2^ = 0.72, AIC = 379.92, *p* < 0.001, *n* = 131), and the performance was superior to other models (Table 2) with a coefficient and exponent of 1.20 and 0.75, respectively.

In this study, we collected 100 cm^2^ circular-shaped EB samples (*n* = 131) from six forest stands across an elevation range of 1,200–1,950 m a.s.l. in Chilan Mountain. The mean (±SD) sampled EB depth and biomass were 4.5 ± 2.9 cm and 0.036 ± 0.0203 g cm^−2^, respectively. Significant positive correlations (*p* < 0.005) were found among EB depth and biomass with different regression models (Table 2). Performance of the allometric equation of the power of variance covariate function (*R*^2^ = 0.72, *p* < 0.0001) with smaller AIC and BIC and greater log likelihood was superior to other models, and the model was selected for further analyses (Fig. 2).

**Table 2:** Model performance comparison of allometric equations by referring to values of the Akaike Information Criterion, the Bayesian Information Criterion and log likelihood. Model performance comparison of allometric equations (*W* = αD^β^ for all models, Eq. (1)) by referring to values of the Akaike Information Criterion (AIC), the Bayesian Information Criterion (BIC) and log likelihood. We note that all models are significant with *p* < 0.001.

Method	Model	α	β	*R*^2^	AIC	BIC	Log likelihood
Nonlinear least squares	Nonlinear squared regression	1.26	0.72	0.72	395.12	403.75	−194.56
	Nonlinear squared regression*	1.00	0.84	0.70	400.34	406.09	−198.17
Generalized nonlinear least squares	Power of a variance covariate	1.20	0.75	0.72	379.92	391.43	−185.96
	Exponential of a variance covariate	1.18	0.77	0.72	380.12	391.62	−186.06
	Constant power of a variance covariate	1.18	0.76	0.70	380.91	395.28	−185.45

**Note:**

* Nonlinear squared regression with the fixed α of 1.00.

### The tree-scale EB biomass estimation

**Figure 3:**
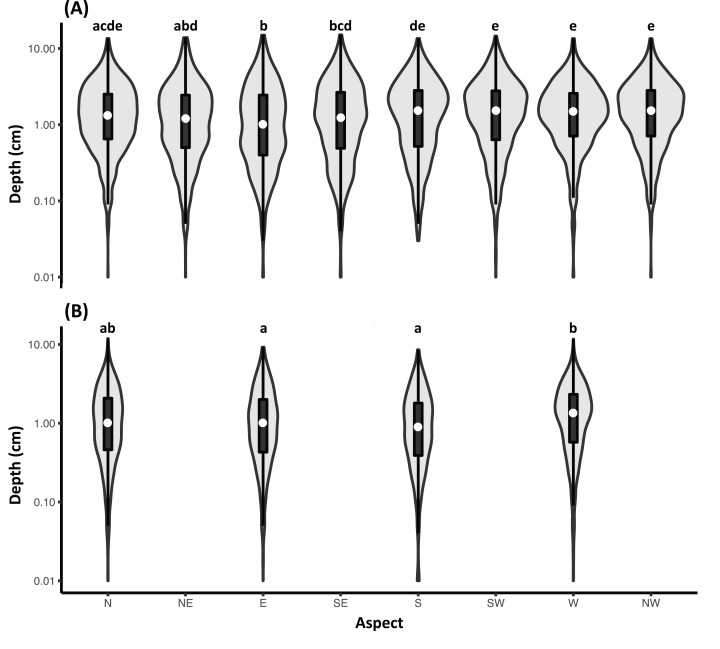
Comparisons of depths of epiphytic bryophytes on sampled stem surface area of 210 sampled trees for different aspect classes. Depths of epiphytic bryophytes (EBs) on the sampled stem surface area of 210 sampled trees for (A) eight (trees with diameter at breast height (DBH) > 20 cm) and (B) four (20 cm ≥ DBH ³ 5 cm) aspect classes, and statistical details can be found in Table S1. The white dots indicate the medians and the thick black vertical lines are the ranges of 25% and 75% percentiles. Pairs of groups with no common capital letters indicate significant differences (*a* ≤ 0.05) by referring to Tukey–Kramer multiple comparisons. We note that the values of EB depth are transformed via the natural logarithm for better visualization.

**Figure 4:**
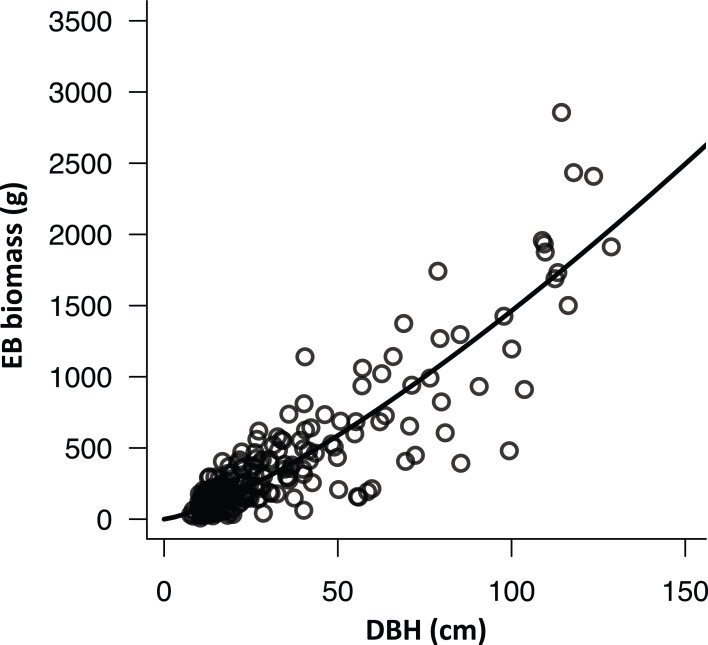
The relationship between diameter at breast height and epiphytic bryophyte biomass of sampled stem surface area. The relationship between diameter at breast height (DBH) and epiphytic bryophyte (EB) biomass of sampled stem surface area based upon 10 sampled trees of different DBH sizes on the 21 field plots (*n* = 210): EB biomass = 3.40 DBH^1.32^ (*R*^2^ = 0.86, *p* < 0.001).

Ten trees evenly distributed along the DBH gradient of each plot (total 210 trees) were selected to investigate the relationship between DBH and BD of EB-hosted trees. The mean (± SD) DBH and BD of sampled trees were 33.5 ± 27.8 cm and 49.5 ± 34.5 cm, respectively. High correlation (*R*^2^ = 0.94, *p* < 0.0001) was found between DBH and BD (Fig. S3). With this information, we computed SSA in the plots by referring to Eqs. (2) and (3). The mean (± SD) of SSA was 3.5 ± 2.8 m^2^. Mean (± SD) EB depth of the 210 sampled trees was 1.1 ± 0.6 cm, and aspect did not affect EB depth greatly with mean (± SD) difference of 0.19 ± 0.12 cm and 0.12 ± 0.07 cm for eight (*n* = 10,296) and four (*n* = 4,092) direction measurements, respectively (Fig. 3; Table S1). However, the EB depths on westward exposures (southwest, west and northwest) were significantly higher (α ≤ 0.05) than those on other aspects according to the Tukey–Kramer multiple comparison. The data was injected into the allometry (Fig. 2) to yield EB biomass density (mean ± SD) of 0.0102 ± 0.0052 g cm^−2^ (or 402.2 ± 478.9 g on SSA). We note that there was a significant positive curvilinear relationship (*R*^2^ = 0.86, *p* < 0.001) between DBH of the sampled tree and EB biomass on SSA (Fig. 4).

**Figure 5:**
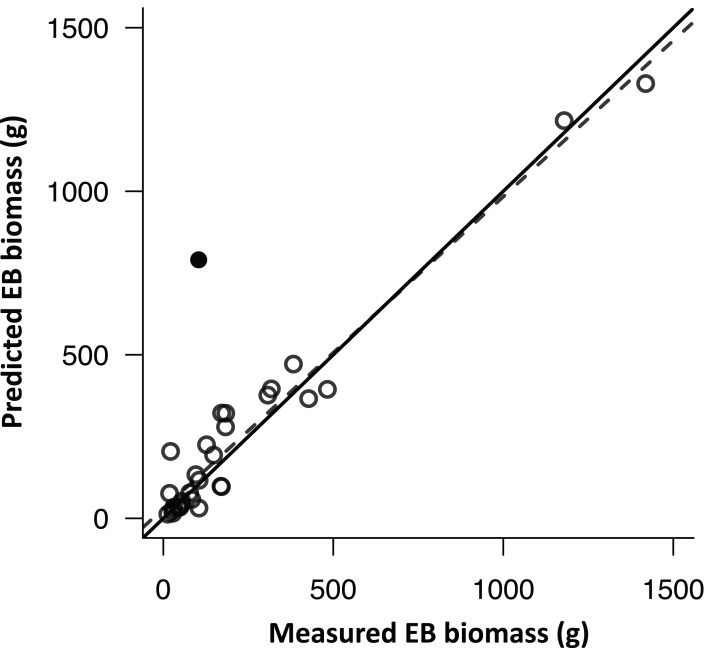
The comparison of model-predicted epiphytic bryophyte (EB) biomass and field-collected EB biomass. The comparison of model-predicted epiphytic bryophyte (EB) biomass and field-collected EB biomass on the sampled stem surface area. The black solid dot indicates an apparent outlier in which EB inhabited on decomposed tree bark. The solid and dashed lines indicate 1:1 and correlation relationships, respectively.

Biomass of epiphytic bryophytes on 30 randomly selected trees with mean (± SD) DBH of 26.2 ± 21.5 cm was destructively collected to verify the proposed approach of upscaling the patch scale estimation (Fig. 2) to SSA. Overall, the performance was satisfactory (Fig. 5) and all samples but one outlier (*R*^2^ = 0.82 and 0.95 without the outlier, *p* < 0.0001 for both model) were close to the 1:1 line (slope = 0.93 and 0.95 without the outlier, *p* > 0.8 for the intercepts of both models) with the mean absolute difference of 77.3 g (35.2% of the mean estimate) or 56.3 g (25.2% of the mean estimate) without the outlier. The outlier may be possibly due to rotten and softened tree bark underneath the EB mats (observed during the sample cleaning), and the depth of tree bark may have been included in the EB depth measurement, resulting in pronounced over-estimation. By applying the in-situ conversion function (Eq. (4)), the EB biomass (mean ± SD) for each sampled tree within the plots was estimated (818.3 ± 1335.1 g) (*n* = 210).

### The plot and regional scales EB biomass estimation

Mean (± SD) DBH of the trees (*n* = 1451; 1,308 hinoki cypress, 139 Japanese cedar and 4 broadleaf trees) within twenty-one plots was 20.3 ± 17.5 cm (detailed plot-scale statistics of forest stands see Fig. S4; Table S2). The EB biomass (and biomass density) for each plot can be interpolated by referring to the EB biomass of 10 sampled trees within each plot with the mean ± SD of 24.5 ± 9.4 kg (or 272.0 ± 104.0 kg ha^−1^). Twenty-one refereed papers were found, and 86% (18/21) of the studies reported higher EB biomass density values than our mean plot/stand scale estimation (Table 1).

## Discussion

Epiphytic bryophytes are some of the most quintessential species characterizing mid-altitude tropical montane cloud forests (Bruijnzeel, Scatena & Hamilton, 2010) and play a pivotal role in influencing the global hydrological cycle (Porada, Van Stan & Kleidon, 2018). Due to the diverse morphology of the species and their “epiphytic” habitat, it is difficult to quantify the abundance of EB. In this study, we propose a novel field protocol for regional EB biomass estimation. Our discussion will mainly focus on (1) EB depth-biomass allometry, (2) scaling of EB biomass from the patch to the plot scale, and (3) limitation and future directions.

### The patch scale EB depth-biomass allometry

In this study, in-situ general (species-unspecific) allometric equations were developed to estimate the biomass of a 100 cm^2^ circular patch of EB using an objective plant structural metric, the central depth of the sample (Fig. 2). Utilization of a single explanatory variable may facilitate efficient sampling. This is crucial since the abundance of EB is sensitive to microclimate governed by the environment (Werner et al., 2012), and the proposed approach can rapidly assess EB biomass in a myriad of physical settings. The performance of the allometric model was satisfactory (Fig. 2; Table 2), even though the morphology of EB is much more diverse than most vascular plants. This answers our first research question (Q_1_) of the statistical significance of the allometric relationship between the patch scale EB biomass density and a single structural parameter (the EB depth).

Plant allometry focuses on relationships between plant body size and biomass, production, population density or other abundance related dependent variables (Enquist, Brown & West, 1998; Enquist et al., 1999). Stanton & Reeb (2016) suggested that some characteristics of bryophytes may be allometrically scaled like vascular plants, which was verified in this study. The mean exponent of the five selected power models was 0.75 (3/4) (Table 2), which agrees with the 3/4 power law (Kleiber, 1947) and is similar to the constant scaling exponents over a wide range of vascular plant size, often with quarter-powers in metabolic scaling theory using biomass as an independent variable (West, Brown & Enquist, 1997, 1999). However, epiphytic bryophytes are non-vascular plants composed of a simple stem, which has a limited role in transporting moisture and nutrients through conducting tissues and does not follow the vascular transport system as a self-similar, fractal-like branching network (Ligrone, Duckett & Renzaglia, 2000). Two major branching forms of bryophytes are sympodial with connected modules of equal level and monopodial (Stanton & Reeb, 2016). For most vascular plants, the branching bifurcation is two (Enquist et al., 2007), and the height is 1/4 exponent of mass (West, Brown & Enquist, 1999). This is inconsistent with our empirical observations, although the sampling unit was a mat but not an individual. This could verify that the basic assumption of an organism’s self-similar branching network plays a major role in governing the allometric relationship.

### Up-scaling of EB biomass

Abundance and distribution of EB may be affected by topography (Chantanaorrapint & Frahm, 2011), and host tree specific (Studlar, 1982) and age (Spitale, 2017) variations. The field survey was conducted across a wide elevation gradient (1,200–1,950 m a.s.l.) in Chilan Mountain, inhabited by relative homogeneous coniferous forest types. In addition, the sampled 21 plots covered old-growth, regenerated and plantation forest stands (Hu & Huang, 2019). Estimation (statistics) of EB biomass may be representative for Chilan Mountain. A point-intercept field instrument (Fig. 1) was invented in this study to facilitate sampling EB height data along a tree stem (SSA, Figs. 4 and 5) based upon the empirical patch scale general depth-biomass allometric model (Fig. 2; Table 2), and later extrapolated to the tree scale using an in-situ conversion equation (Eq. (4)). This responds to our second research question (Q_2_) about the possibility of upscaling the EB patch scale biomass estimation to the whole tree scale.

The distribution of EB biomass on a tree could be very sensitive to the ambient environment and microclimate (McCune, 1993; Sillett & Antoine, 2004). Therefore, we measured the depth of EB in four and eight directions for small (DBH ≤ 20 cm) and large (DBH > 20 cm) trees, respectively, which may reduce microclimate-induced biases. According to Teng (2006), about 74% of EB biomass was present on SSA (Fig. S2). The differences (≤ 0.43 cm) in EB heights (also biomass, Fig. 2) among aspect groups on SSA were biologically insignificant (Table S1). Although statistics showed the EB heights of some aspect groups were different to other groups (Fig. 3), those were possibly due to large sample sizes making error bars negligible. Therefore, we found that abundance of EB in this humid region was more sensitive vertically along the height of the tree than horizontally in the study region.

The proposed sampling method was efficient, taking about 15 min for the four-direction measurement and double that amount of time for the eight-direction measurement. This may permit rapid sampling to obtain a large sample size (Table 1). With proper sampling design and data inter/extrapolation, we may be able to estimate EB biomass in a large region. This replies to the first part of our third research question (Q_3_) regarding to the feasibility of the proposed sampling procedures for the plot-scale EB biomass estimation. Mean biomass density of EB estimated in this study was similar to the one conducted in the same region (230 kg ha^−1^) but within a much smaller spatial extent using a destructive tree harvesting approach (Teng, 2006). Our mean plot (forest stand) scale estimation of EB biomass density falls within the lower half of the EB biomass density global synthesis data (Table 1). We note that it is challenging to make a fair comparison since those previous studies were conducted using different data collection methods over a wide range of geographical extents. In addition, amount of trees being sampled could also play some role in affecting the estimation. However, in terms of efficiency, the proposed new approach is indeed superior to other methods for the sampling of 210 host trees in this study.

This point-intercept approach should also be applicable for the estimation of ground bryophyte biomass, and facilitates the estimation of overall abundance of bryophytes in an ecosystem. This is a pivotal but rarely available parameter, and has a major impact on regulating the terrestrial hydrological cycles (Porada, Van Stan & Kleidon, 2018). This study focused on the height of a tree below 300 cm from the ground, where the majority of EB are present (Trynoski & Glime, 1982) (Fig. 1B). The sampled stem area may be further extended with aid of a foldable ladder.

### Limitation and future directions

One potential research limit is that the tree scale EB biomass estimation, which was extrapolated from the estimation on SSA (Eq. (4)), could not be validated with empirical data. The task is rather difficult and may be impractical for the study region. It requires tree climbing or destructive tree harvesting to strip EB of an entire tree. However, the support of tree climbing was not available during the time of conducting this study, and it could be unsafe to climb a small-size tree without reliable support for a climber’s body weight. Logging for both natural and plantation forests has been completely forbidden in Taiwan since 1991. Therefore, the latter option may not be possible due to the local regulation. In the future, we might be able to take the advantage of tropical cyclone-induced fallen logs and harvest EB biomass at the ground level, since the island is located in a typhoon-prone region (Chi et al., 2015). However, this sampling approach could be biased since the probability of the strong wind induced tree falling may be associated with topography (Mitchell, 2013), which also plays a pivotal role in governing the abundance of EB (Werner et al., 2012). Finally, the proposed field sampling approach may not be practical for settings where the majority of the epiphytes are present in the upper layer of trees (Komposch & Hafellner, 2000). Tree climbing or harvesting may still be necessary and the challenge of field sampling remains.

It is extremely difficult to non-destructively measure EB biomass, and a new field approach was developed in this study to tackle this task. This is crucial because the age of EB on a tree could be almost as old as the age of the host tree (Kimmerer, 2003), and it may require many years of recovery after the removal of samples (Fenton, Frego & Sims, 2003). It may be useful to further generalize the EB allometry (see Data S1) by combining data of different regions to make it applicable for other settings. According to this study (Fig. 4) and some previous literature (Hsu, Horng & Kuo, 2002; Köhler et al., 2007; Chen, Liu & Wang, 2010), we found that there may be a significant relationship between tree size and the abundance of EB (Gómez González et al., 2017). With the availability of a three-dimensional tree size spatial layer at the regional scale derived from high spatial resolution airborne lidar (light detection and ranging) or aerial photographic point cloud data (Chung et al., 2019; Kellner et al., 2019), we may be able to map wall-to-wall EB biomass over a vast region. This also answers the second part of our third research question (Q_3_) as to the potential of the proposed EB biomass measuring approach for the regional scale spatial assessment.

## Conclusion

The biomass of EB is pivotal in governing the forest water and nutrient cycles in TMCFs. However, quantification has been very challenging by nature. This study develops an allometric scaling approach to estimate EB biomass at the individual tree and plot scales. Overall, the performance of the proposed approach was satisfactory. Since a significant relationship between tree size and EB abundance is commonly found in TMCFs, regional EB biomass may be assessed by integrating this field method and three-dimensional high-resolution airborne data.

## Supplemental Information

10.7717/peerj.9351/supp-1 Supplemental Information 1 Supplemental Figures and Tables.

10.7717/peerj.9351/supp-2 Supplemental Information 2 The raw data used to plot Figs. 2-5 and S2.

## Additional Information and Declarations

### Field Study Permissions

The following information was supplied relating to field study approvals (i.e., approving body and any reference numbers): Field experiments were approved by the Veterans Affairs Council, R.O.C. (#1080002884).

### Data Availability

The following information was supplied regarding data availability: The raw data is available in Data S1.
