# Supplementary material for: Epiphytic bryophyte biomass estimation on tree trunks and upscaling in tropical montane cloud forests
Source: PeerJ. 2020 Jun 12;8:e9351. doi: 10.7717/peerj.9351 (PMC7295022; doi:10.7717/peerj.9351)
Supplement: Supplemental Information 1 [file peerj-08-9351-s001.docx]

**Supplementary Information**

**Epiphytic bryophyte biomass estimation on tree trunks and upscaling in tropical montane cloud forests**

Guan-Yu Lai^1^, Hung-Chi Liu^1^, Ariel J. Kuo^2^, Cho-ying Huang^1,3*^

^1^Department of Geography, National Taiwan University, Taipei 10617, Taiwan

^2^Department of Civil and Environmental Engineering, University of California, Los Angeles, CA 90024, USA

^3^Research Center for Future Earth, National Taiwan University, Taipei 10617, Taiwan

^*^Corresponding author

Tel: 886 2 33663733; E-mail: choying@ntu.edu.tw


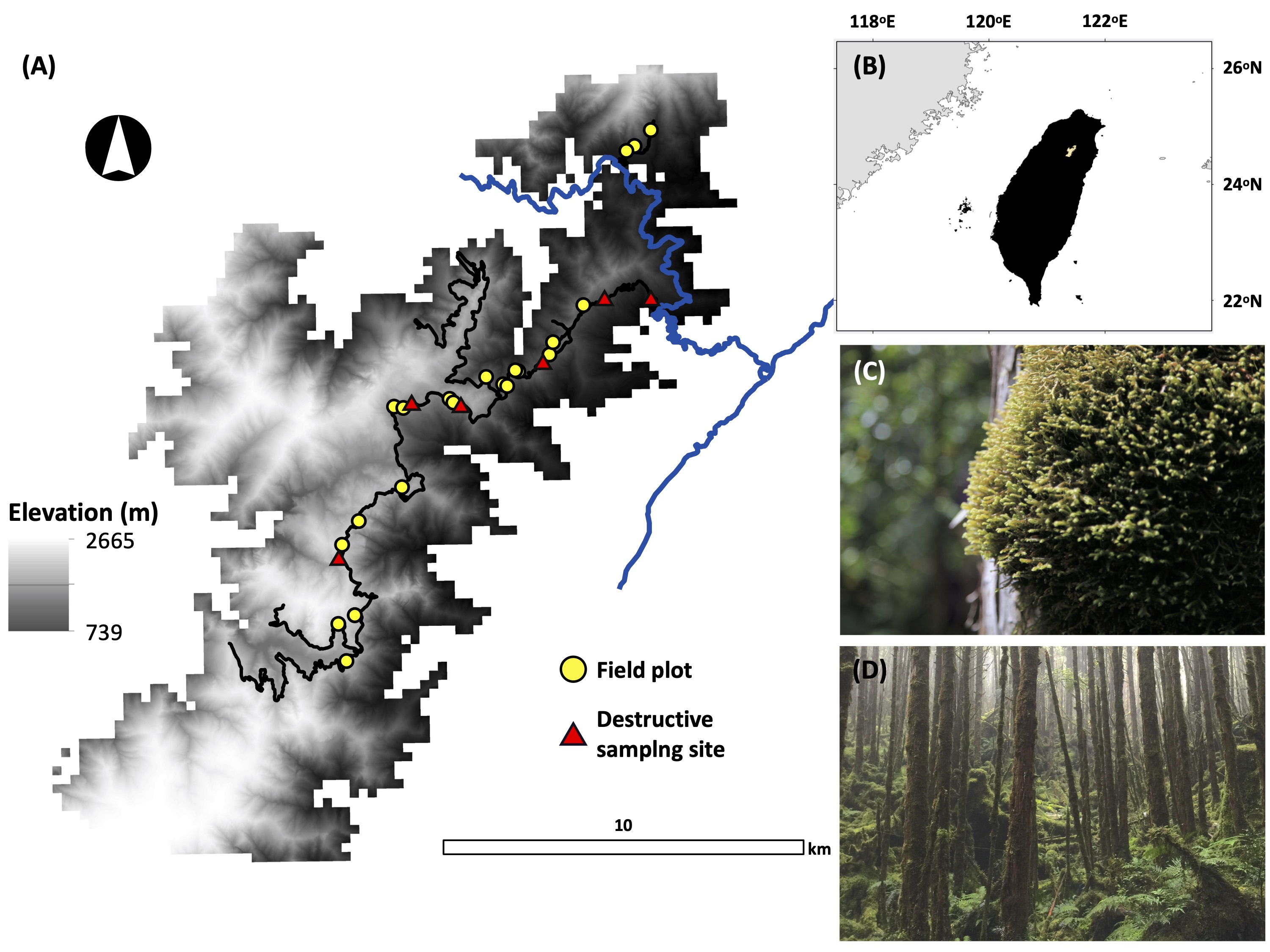


**Fig. S1** (A) The study area (24^o^98’N, 120^o^97’E), tropical montane cloud forests in Chilan Mountain, located in (B) northeast Taiwan (the yellow polygon). The blue and black colored lines are the state and unpaved forest routes, respectively. The gray monochromatic colored background is the 30 m ASTER (the Advanced Spaceborne Thermal Emission and Reflection Radiometer) Global Digital Elevation Model data. Epiphytic bryophyte (EB) mat samples (n = 131, Fig. 2) were destructively collected from six forest stands (red triangles) to develop depth-biomass allometric models to estimate EB biomass in twenty-one 30 × 30 m plots (yellow dots) along the elevation gradient. (C) *Bazzania* spp. is one of the dominant EB species found in (D) this tropical montane cloud forest. The photographs were taken by GY Lai in summer 2017.


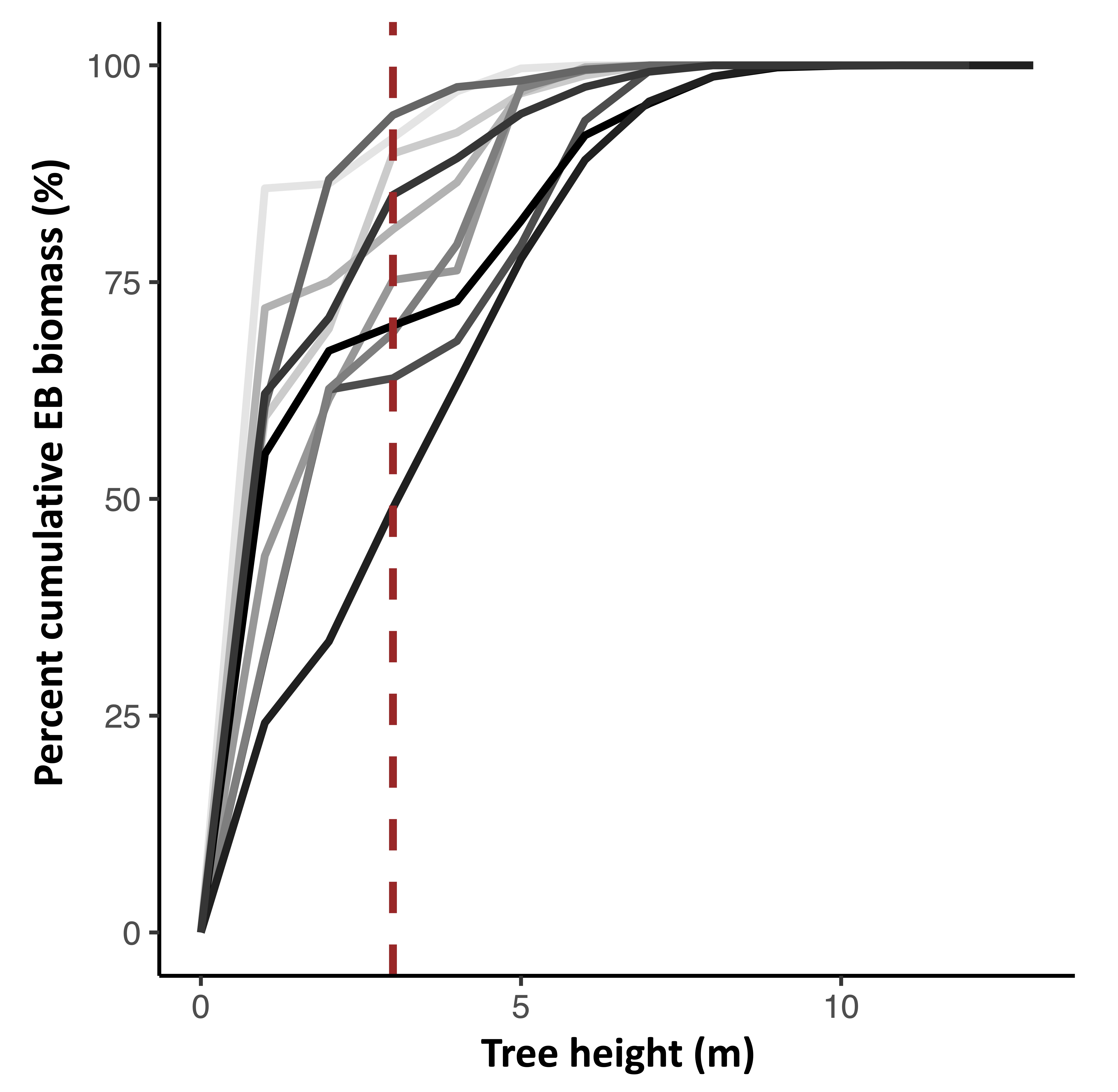


**Fig. S2** The relationship between percent cumulative epiphytic bryophyte (EB) biomass and the tree heights of 10 hinoki cypress sampled in Teng (2006). The monochromatic grayscale colors indicate tree heights from the shortest (light gray, 7.1 m) to the tallest (black, 12.8 m) trees. The red dashed line indicates the 3-m height above the ground, and EB depths below this height within 21 field plots (Fig. S1) were recorded using a newly developed instrument (Fig. 1).


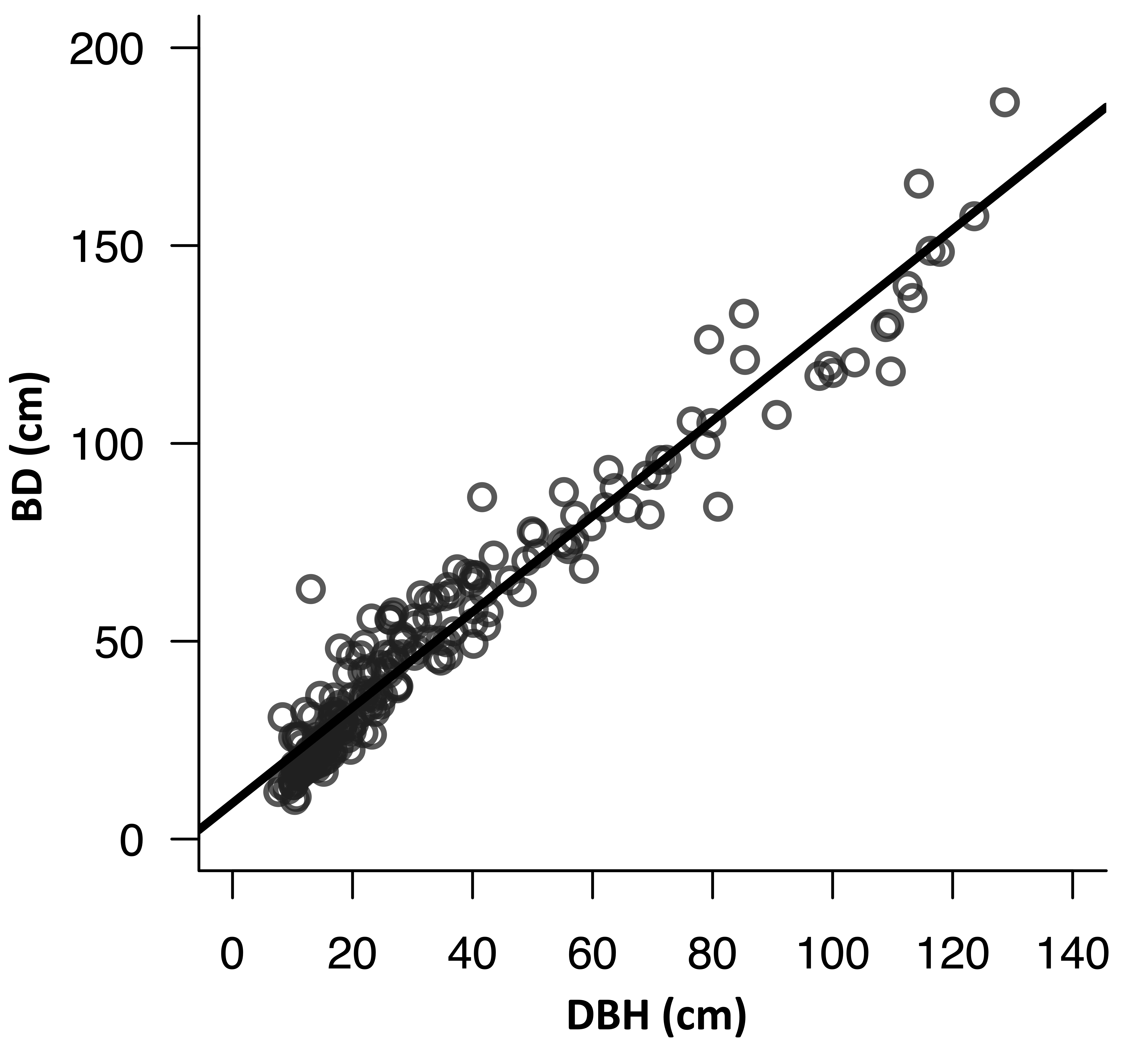


**FIG. S3** The relationship between diameter at breast height (DBH) and basal diameter (BD) of 210 sampled trees within twenty-one field plots (BD = 9.1 + 1.2DBH, R^2^ = 0.94, *p* < 0.001).


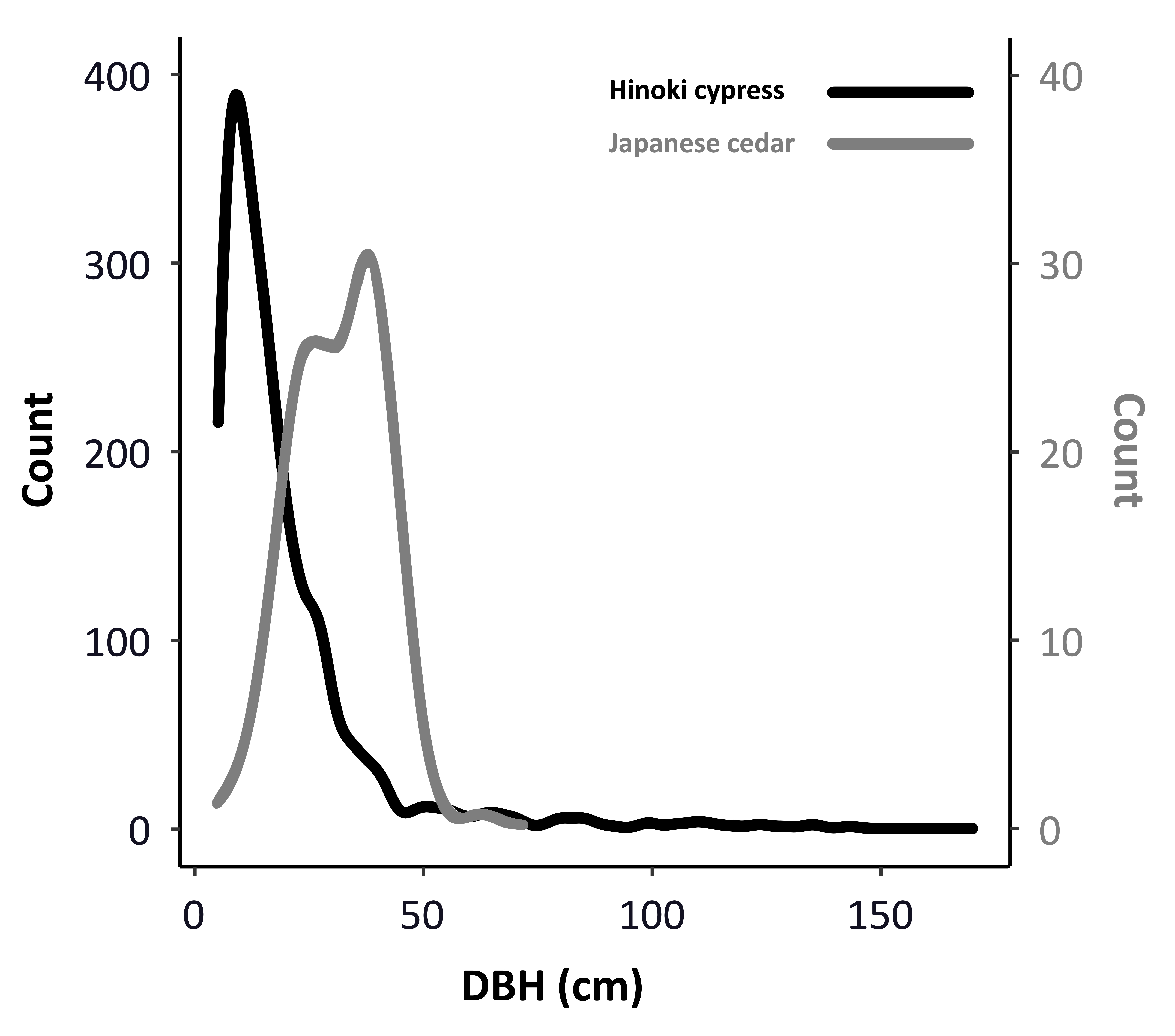


**FIG. S4** Distributions of diameter at breast height (DBH) of hinoki cypress (n = 1308, the black smoothed line) (the primary y-axis) and Japanese cedar (n = 139, the gray smoothed line) (the secondary y-axis) in the 21 plots (Fig. S1). Due to the small sample size (n = 4), the data of broadleaf trees are not reported here.

**TABLE S1** Statistics (mean, standard deviation [SD], minimum [min], maximum [max]) of depths (cm) of epiphytic bryophyte mats on sampled stem surface area below 3 m of 117 large (diameter at breast height [DBH] > 20 cm) and 93 small (20 cm ≥ DBH ≥ 5 cm) trees. Pairs of groups with no common superscript capital letters (a-e) indicate significant differences (α ≤ 0.05) by referring to Tukey-Kramer multiple comparisons.

| Aspect group | Mean (SD) | Min–Max |
| --- | --- | --- |
| *Eight directions (n = 10296)* |  |  |
| North | 1.21 (1.72)^acde^ | 0.00–13.56 |
| Northeast | 1.12 (1.87)^abd^ | 0.00–13.89 |
| East | 0.98 (1.83)^b^ | 0.00–14.99 |
| Southeast | 1.03 (1.80)^bcd^ | 0.00–15.12 |
| South | 1.21 (1.81)^de^ | 0.00–13.56 |
| Southwest | 1.41 (1.90)^e^ | 0.00–14.60 |
| West | 1.37 (1.74)^e^ | 0.00–13.50 |
| Northwest | 1.34 (1.79)^e^ | 0.00–13.50 |
| *Four directions (n = 4092)* |  |  |
| North | 0.87 (1.30)^ab^ | 0.00–11.90 |
| East | 0.83 (1.32)^a^ | 0.00–9.23 |
| South | 0.78 (1.19)^a^ | 0.00–8.56 |
| West | 1.00 (1.35)^b^ | 0.00–11.67 |

**TABLE S2** Statistics (mean, standard deviation [SD], minimum [min], maximum [max]) of twenty-one 30 × 30 m forest stand characteristics (count, density, diameter at breast height [DBH] and sampled stem surface area below 3 m [SSA]). There were 1308 hinoki cypress, 139 Japanese cedar and 4 broadleaf trees.

|  | Mean (SD) | Min–Max |
| --- | --- | --- |
| Count | 69.1 (46.3) | 9.0–208.0 |
| Density (# ha^-1^) | 767.7 (514.9) | 100.0–2311.0 |
| DBH (cm) | 28.6 (18.8) | 5.0–175.0 |
| SSA (m^2^) | 143.6 (58.6) | 62.6–284.1 |

**REFERENCE**

Teng, C.-H. 2006. The composition, distribution, and biomass of epiphytic bryophytes of a naturally regenerated Chamaecyparis obtusa var. formosana forest. *Master Thesis*, Department of Natural Resources and Environment Studies, College of Environment Studies, National Donghua University, Hualien, Taiwan.
